# Skeletal muscle cell-specific differences in type 2 diabetes

**DOI:** 10.1007/s00018-022-04265-7

**Published:** 2022-04-23

**Authors:** Noni T. Frankenberg, Shaun A. Mason, Glenn D. Wadley, Robyn M. Murphy

**Affiliations:** 1 Department of Biochemistry and Chemistry, La Trobe Institute for Molecular Science, School of Agriculture, Biomedicine and Environment, La Trobe University, Melbourne, 3086 Australia; 2 Institute for Physical Activity and Nutrition, Deakin University, Burwood, 3125 Australia

**Keywords:** Single fibres, Glycogen, Glucose regulation, Hyperinsulinaemic euglycaemic clamp

## Abstract

Major stores of glucose are found as glycogen in skeletal muscle and liver. Skeletal muscle is a heterogenous tissue, with cellular metabolic and contractile distinctions dependent on whether the cell (fibre) is slow-twitch (Type I) or fast-twitch (Type II). We hypothesised that proteins important for glycogen metabolism would be differentially abundant between these diverse fibres. We further hypothesised that the cellular location of these proteins would be different in muscle samples between control (CON) and individuals with type 2 diabetes (T2D). We dissected individual muscle fibre segments from vastus lateralis skeletal muscle biopsy samples from CON and T2D and used cell-type-specific approaches to address muscle heterogeneity. We measured glycogen and glycogen-related proteins by immunoblotting techniques. A lower proportion of Type I fibres was found in muscle in T2D compared with CON. AMPK-β2, glycogen branching enzyme (GBE), glycogen debranching enzyme (GDE), and glycogen phosphorylase (GP) were differentially localized between fibre types and in fibres from CON and T2D individuals. A key novel finding was that the majority of glycogen is loosely bound or cytosolic in location in human skeletal muscle. The proportion of this diffusible pool of glycogen was significantly lower in Type I fibres in T2D compared to CON. A hyperinsulinaemic, euglycaemic clamp in people with type 2 diabetes had no effect on the proportion of diffusible glycogen. We identify cell-type as an important consideration when assessing glycogen metabolism in muscle. Our findings demonstrate varying glucose handling abilities in specific muscle fibre types in type 2 diabetes. A model is presented to provide an overview of the cell-specific differences in glycogen metabolism in type 2 diabetes.

## Introduction

Skeletal muscle is critical for maintaining whole body glucose homeostasis and impaired glucose regulation can result in type 2 diabetes [1]. Skeletal muscle is a heterogeneous tissue composed of different cell (fibre) types classified according to the specific myosin heavy chain isoform (MHC) present. Type I (slow-twitch) and Type II (fast-twitch) fibres are distinct in their contractile and metabolic characteristics [2]. Studies have indicated a fibre type dependent rate of insulin-stimulated glucose uptake with a higher glucose uptake related to the oxidative capacity of the muscle fibre (Type I << IIa < IIx < IIb) in both rats [3–5] and humans [6]. Furthermore, several studies in humans have demonstrated positive correlations between the proportion of Type I fibres in muscle and whole-body insulin sensitivity [6–8]. Together these findings indicate that human Type I fibres have a greater influence in maintaining glucose homeostasis than Type II fibres. Furthermore, a reduction in the proportion of Type I fibres in muscle from patients with type 2 diabetes has been observed in several [8–11], but not all [6, 12] studies.

There are several key signalling proteins and enzymes vital to the regulation of glucose metabolism. Alteration in abundance or regulation of these could be a driving factor leading to fibre-type-specific differences in glucose regulation. Glycogen is a branched polymer of glucose and is the main glucose source within contracting skeletal muscle. Glycogen synthesis is initiated by the auto-glycosylation of glycogenin and elongated by the activities of glycogen synthase (GS) and glycogen branching enzyme (GBE) [13]. Glucose is mobilized from glycogen by the concerted action of glycogen phosphorylase (GP) and glycogen debranching enzyme (GDE) acting in reverse to GS and GBE [14]. In rat skeletal muscle the abundance of GDE and GP was higher in type II dominant muscle whereas GBE abundance was higher in Type I dominant muscle and GS showed no fibre type dependency [15, 16]. Given this direct influence glycogen has on glucose metabolism, it suggests the abundance of glycogen-related proteins and their involvement in glycogen synthesis and degradation may differentially impact glucose regulation in different fibre types. It is feasible to think intracellular glycogen metabolism may be different between muscle fibre types given that type 2 diabetes results in differences in proteins important for glucose regulation such as AMPK (see below) and that these proteins are differentially expressed in muscle fibre types. Using mass spectrometry, it has been shown that proteins related to glycogen metabolism are fibre type dependent in human skeletal muscle, although details for which specific proteins were not presented [17]. The movement of glycogen-related proteins have yet to be elucidated in human muscle fibres.

AMPK is widely regarded as a super metabolic regulator and is reported to regulate and/or interact with glycogen-related proteins and GLUT4 [18]. We have previously reported AMPKβ1 levels are higher in Type I compared with Type II human muscle fibres [19]. However, it is unknown if this translates to differences in the how fibre types respond to insulin. In addition to the abundances of proteins, their localization is an important factor influencing cellular processes and, in this case, glucose regulation. It has been suggested that the AMPKβ isoforms are membrane bound in human skeletal muscle [20], although in rat skeletal muscle we have reported that ~ 75% of the total AMPK pool is diffusible and thus likely of cytosolic localisation [16]. Regardless of its localisation in resting skeletal muscle, AMPK phosphorylation may result in its movement in muscle and hence regulation [21]. In addition, it is possible that any dysregulation in the ability for AMPK to move within muscle may result in biologically relevant alterations in glycogen regulation which could be a confounding factor in type 2 diabetes.

The major aims of this study were to examine the fundamental fibre-specific abundance and intracellular localization/diffusibility of several key glycogen-related proteins in healthy individuals and people with type 2 diabetes, and to determine whether increased insulin, administered as a hyperinsulinaemic, euglycaemic clamp, alters this expression in skeletal muscle from people with type 2 diabetes. This was achieved by examining single fibres expressing either MHC I or II dissected from human vastus lateralis biopsies from control and people with type 2 diabetes; the latter at rest and following a hyperinsulinaemic, euglycaemic clamp.

## Research design and methods

### Participants

Eleven control (CON) and type 2 diabetes (T2D) participants are included, who were involved in two separate previously published studies conducted at Deakin University [22] and Victoria University [23], both universities being located in Melbourne, Victoria. Samples were collected over a 5-month period. All samples were taken at rest during the morning. Both studies were approved by the appropriate ethics committee and carried out in accordance with the Declaration of Helsinki II. CON individuals (8 male and 3 female) mean (± SD) age 62 ± 13 years, height 170 ± 9 cm and weight, 74 ± 12 kg and no medical diagnosis of type 2 diabetes. T2D individuals (10 male and 1 female) mean (± SD) age was 59 ± 9 years, height 172 ± 0.1 cm, weight 90 ± 12 kg, diabetes duration 5.4 ± 5 years. Metabolic characteristics of the type 2 diabetes participants have been published, and all were from the placebo trial [22]. A limitation of the study was the inclusion of males and females; however this was unlikely to be a concern because comparison of cells derived from males and post-menopausal women showed no intrinsic differences in glucose metabolic pathways [24].

### Muscle biopsy

Experiments were performed with tissue obtained from a thigh muscle (vastus lateralis) biopsy in rested conditions. After injection of a local anaesthetic (1% Xylocaine) into the skin and fascia, a small incision was made in the middle third of the vastus lateralis muscle of each subject and a muscle sample taken using a Bergstrom biopsy needle modified to include suction. An experienced medical practitioner took all biopsies at approximately constant depth and the second biopsy was taken approximately 1–2-cm proximal to the previous sampling site. Muscle biopsies were collected at rest (CON) before (Pre T2D) and 60 min after commencement of the 40 mU/m^2^/min hyperinsulinemic euglycemic clamp (Post T2D) that raised plasma insulin levels to 58 ± 9 µU/ml (Mean ± SD) [22]. Excised samples of ~ 100–200 mg were blotted on filter paper to remove excess blood and a portion (~ 5–10 mg) placed in paraffin oil (Ajax Chemical, Sydney, Australia) for fibre dissection.

### Fibre segment preparation

The muscle biopsy was pinned at resting length in a Petri dish on a Sylgaard layer under paraffin oil. Individual fibre segments were dissected from the biopsy under a microscope at room temperature (RT), typically 2–3 mm in length which is equivalent to ~ 10–15 µg wet weight muscle [16, 25, 26]. The same experimenters collected fibres from all biopsies. To determine protein abundance in a given fibre type, isolated muscle fibre segments were individually placed into 1 × SDS loading buffer, consisting of 10 µl physiological buffer (PB) (in mM; 129 K^+^, 1 free MG^2+^ (10.3 total Mg^2+^), 90 HEPES, 50 EGTA, * ATP, 10 CP, pH 7.10 and an osmolality of 295 ± 10 mosmol/kg H_2_O) and 5 µl 3 × SDS loading buffer (0.125 M TrisHCl pH6.8, 4% SDS, 10% glycerol, 4 M urea, 10% mercaptoethanol and 0.001% bromophenol blue). The proportion of a protein in the cytosol was assessed using our protein diffusibility assay, using mechanically-skinned fibres by rolling back the surface membrane (sarcolemma) using fine forceps, as described previously [16, 26]. Briefly, skinned fibre segments were tied with silk suture and immersed into 10 µl wash PB with protease inhibitor cocktail (COMplete; Roche Diagnostics, Sydney, NSW, Australia) for 10 min allowing any freely diffusible proteins (i.e., cytoplasmic proteins) to move out of the fibre. We performed a diffusibility time course assay with 1-, 10- and 30-min timepoints for all proteins except GS, due to the inability to probe for that protein after GP as they have similar migrations on a gel and GP at 30 min. Importantly, this assay does not include any manipulation of the sample, such as via vortex or centrifugation, ensuring only the proteins with a cytosolic or loosely bound location to be identified. The fibre was then removed from the wash solution and placed into a new tube with 10 µl PB. Finally, 5 µl 3 × SDS loading buffer was added to all tubes, which were stored at − 80 °C until analysed by western blotting, where the wash and fibre were run side-by-side.

### Western blotting

Whole protein muscle samples were separated on 4–15% Criterion Stain Free gels (Bio-Rad, Hercules, CA) as previously described [19, 27, 28]. A mixed sample was loaded onto gels (2, 4, 8 and 16 µl) to generate calibration curves to determine detection limits for a given protein and comparison across gels. Total protein in the gel were imaged using Stain Free imager (Bio Rad). Loading amount was measured by determining the total density of all the bands in each lane. Following transfer, nitrocellulose membranes were blocked and probed with the required primary antibody constantly rocked overnight at 4 °C and 2 h at room temperature (rabbit anti-AMPKβ1/2 1:1000 Cell Signalling Cat#4150 lot 003, rabbit anti-GBE 1:5000, anti-GDE 1:1000 and anti-GP 1:1000 previously described [29], rabbit anti-GS 1:2500 Epitomics Cat#1919-1, mouse anti-MHCI 1:200 Developmental Studies Hybridoma Bank (DSHB) A4.840, mouse anti-MHCIIa 1:200 DSHB A4.74). Membranes were cut into sections containing proteins of a particular size to maximise the number of proteins that could be detected with first probe of the membrane. Following washes and probes with secondary antibodies (goat anti-mouse IgG-horse radish peroxidase (HRP) Pierce 31430 and goat anti-rabbit IgG-HRP Pierce 31460, both diluted 1:60,000) chemiluminescent images were captured and densitometry was performed (Chemidoc MP and ImageLab software, version 6, Bio-Rad). Images of the molecular weight markers on the membrane were captured under white light prior to chemiluminescent imaging without moving the membrane.

### Dot blotting for glycogen analysis

Fibres were firstly examined for fibre type by dot blotting as previously described [30]. Succeeding this, fibres and washes were pooled according to their MHC for each individual and treatment. Pooled washes and matched fibres were loaded in triplicate and dot blotted for Glycogen (gift from Otto Baba, Tokyo Medical and Dental University, Tokyo, Japan) along with a standard curve of a muscle homogenate with a known glycogen content [21]. Chemiluminescent images were collected, and densitometry performed using Chemidoc MP and ImageLab software.

### Statistical analyses

Results are presented as mean values + standard deviation (SD) unless otherwise indicated. Protein data were analysed using a two-way, within subject ANOVA, with subject (CON and T2D) and fibre type (Type I and Type II) post-hoc testing (Dunnet’s or Tukey) performed when statistically significant interactions were detected with ANOVA. The *P* values are indicated, and significance was set at *P* < 0.05. Fibre type data were tested using one-way ANOVA with Bonferroni post-hoc analysis. Repeated measures were determined not to be significant using linear mixed model analysis of estimation of covariance parameters for all data sets. This test justifies and increases the power of the data set by permitting the use of fibres as individual data points rather than using the average of all fibres collected for that participant. The amount of a given protein in muscle homogenates was expressed relative to total protein (from density of all bands in that lane of the StainFree gel). For a given treatment and to allow comparison across gels, values were expressed relative to the average of the Type I fibres on the given gel. In addition, the amount of protein in washes obtained from single fibres was expressed as percentage of total pool (wash and fibre) of the protein of interest. The amount of glycogen was determined from the sum of wash and fibre compared to a calibration curve of known glycogen amount. Glycogen data were analysed using univariant ANOVA with a Bonferroni post hoc tests. Time course analyses were analysed using one-way ANOVA within population with Bonferroni post hoc tests. Analyses used Prism version 7.0 (GraphPad) or SPSS Statistics 24.

## Results

### Fibre type composition

**Fig. 1:**
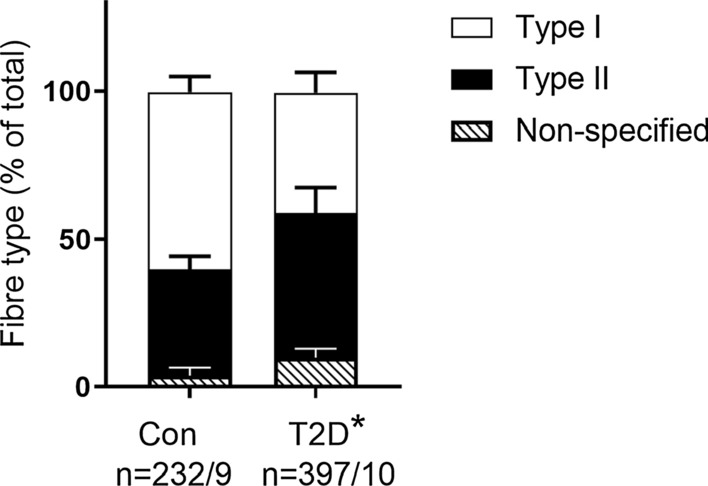
Fibre type composition in control and T2D individuals. Single muscle fibre segments were analysed by Western blot for the presence of either MHCI (white bars, representing Type I fibres) or MHCII (MHCIIa or hybrid MHCIIa/MHCIIx) (black bars, representing Type II fibres) and hybrid MHCI/MHCII or MHCIIx (diagonal stripes bars, representing non-specified fibre type). Data shown as percentage of total composition and SEM. A one-way ANOVA with Bonferroni post hoc test revealed differences in the number of Type 1 fibres (*P* = 0.005) and Type II fibres (*P* = 0.03) between CON and T2D, *n* = number of fibre segments/subjects

MHCI and IIa fibres constituted ~ 60% and 36%, respectively, in CON and 41% and 49%, respectively, in T2D, with non-specified fibres amounting to 4% and 10%, respectively, in CON and T2D (see below). The relative proportion of Type I muscle fibres was lower and Type II muscle fibres higher, in T2D compared with CON (Fig. 1, *P* = 0.005 and *P* = 0.03, respectively, one-way ANOVA with Bonferroni’s post hoc test). Fibres expressing both MHCI and IIa, those that did not stain with MHCIIa antibody and those that were MHCIIx positive (129 fibres in total probed with MHCIIx) were pooled as non-specified fibres types (Fig. 1). There were 76 fibres excluded from further analyses, 23 fibres from CON and 53 fibres from T2D.

### Abundance and fibre-type specificity of glycogen-related proteins and AMPKβ2 in muscle fibres from control and type 2 diabetes individuals

**Fig. 2:**
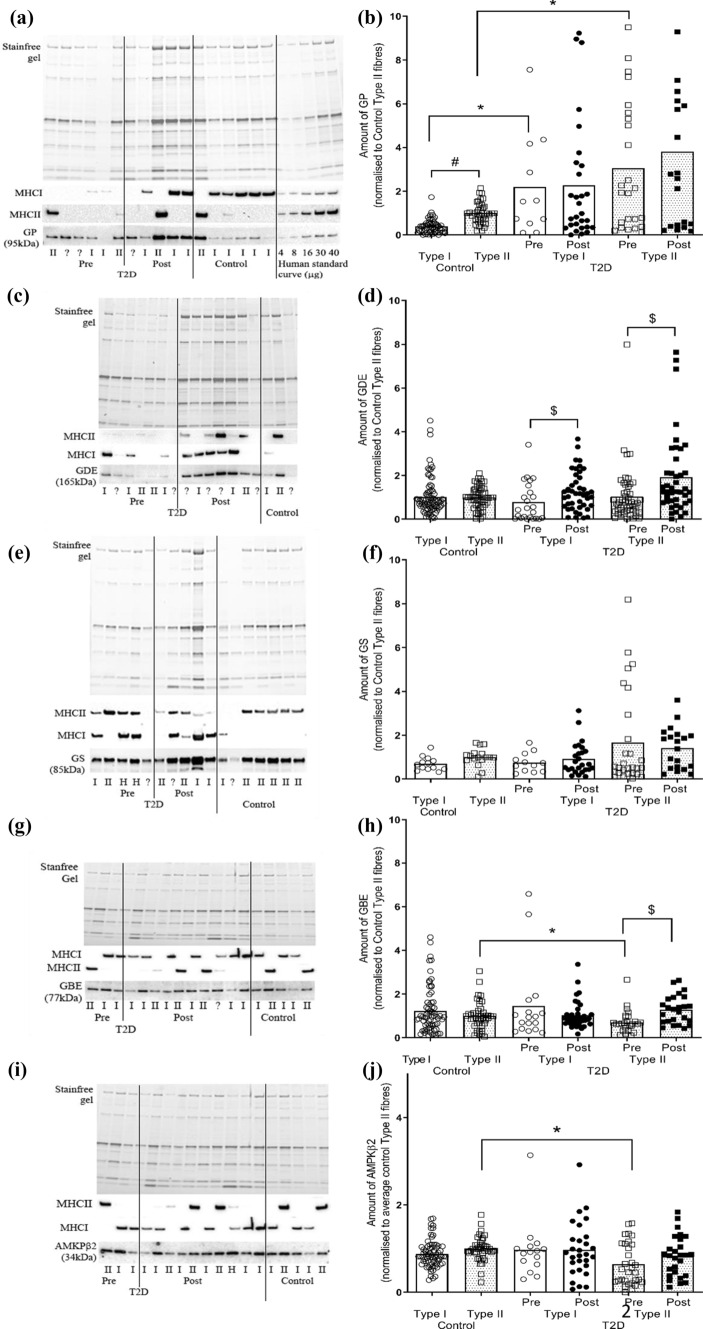
Fibre type dependence of glycogen-related proteins in control and T2D individuals. Amount of glycogen phosphorylase (GP), glycogen debranching enzyme (GDE), glycogen synthase (GS), glycogen branching enzyme (GBE) and AMPK-β2 in Type I and Type II fibres isolated from control (CON) and T2D individuals (before (pre) and after (post) hyperinsulinaemic, euglycaemic clamp). **A** Western blot showing detection of GP, MHC I, MHCII protein in single fibres segments isolated from CON and T2D pre and post clamp and a calibration curve of mixed human muscle homogenates loaded at 4–40 μg wet weight muscle. **B** Mean data expressed relative to the average GP amount in Type II fibres from CON on the given gel (mean + S.D). **C**, **D** As for A and B, but showing GDE. E and F. As for A and B, but showing GS. **G**, **H** As for A and B, but showing GBE. **I**, **J** As for A and B, but showing AMPK-β2. Note this Western blot was also probed with GBE as seen in panel G. Two-way within subject ANOVA. ^#^indicates significant difference (*P* < 0.05) compared to Type I fibres from the same group. * indicates significant difference (*P* < 0.05) compared to the same type of control fibres. $ indicates significant difference (*P* < 0.05) compared to the pre fibres. **A**–**D**, *N* = 11/12–2 individuals/fibres; **E**–**H**, *N* = 4–11/12–2 individuals/fibres; **I**–**J**, *N* = 7–11/12–2 individuals/fibres. ? indicates fibre type not ascertained and through a process of elimination, likely a MHC IIx fibre. Contiguous lanes on a given western blot are shown, with vertical lines included to separate the samples from the different groups as indicated

GP was higher in Type II compared with Type I fibres in CON (*F*_1,6_ = 12.5, *P* = 0.001 univariant, *P* = 0.04 Tukey’s post hoc), but there were no fibre type differences detected in T2D muscle, either before or after the clamp (Fig. 2A, B). Compared with fibres obtained from CON, there was higher GP abundance in both Type I (*P* = 0.02) and Type II (*P* = 0.01) fibres in muscle from T2D. There was no difference in GP in either Type I or Type II fibres obtained before and after the clamp compared to pre (Fig. 2A, B). Whilst no fibre type differences were revealed in GDE from CON or T2D, there was increased GDE following the clamp in Type II fibres in T2D (*F*_1,6_ = 5, *P* = 0.001 univariant, *P* = 0.006 Tukey’s post hoc) (Fig. 2C, D). GS had no fibre type differences in muscle from CON or T2D, no differences between before or after the clamp, or compared with CON (Fig. 2E, F). Whilst no fibre type differences were revealed in GBE from CON, there was a lower GBE abundance in Type II compared with Type I fibres in T2D (*F*_1,6_ = 3.75, *P* = 0.06 univariant, *P* = 0.04 Tukey’s post hoc), and following the clamp, GBE was increased in Type II fibres, with no effect detected in Type I fibres (*P* = 0.05 Tukey’s post hoc) (Fig. 2G, H). AMPK-β2 was higher in Type II from CON compared with T2D (*F*_1,6_ = 2.13, *P* = 0.06 univariant, *P* = 0.04 Tukey’s post hoc), with no fibre type differences detected in CON, and no effect of the clamp in T2D for a given fibre type (Fig. 2I, J).

### Protein movement and localisation in human single fibres from control and T2D individuals

**Fig. 3:**
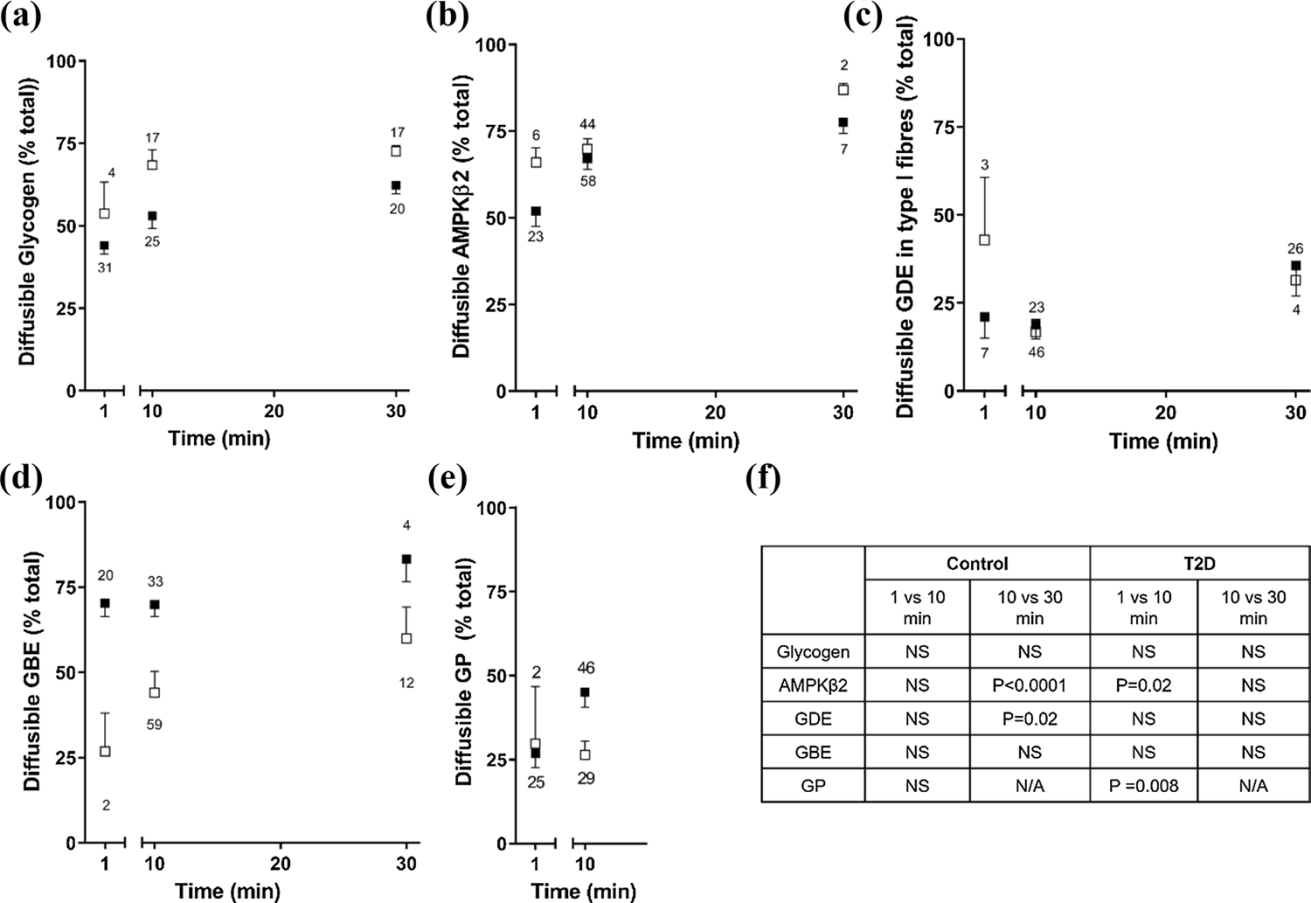
Time course of proportion of diffusible protein in muscle fibres from control and T2D individuals. Diffusion of glycogen (**A**), AMPK-β2 (**B**), and glycogen-related proteins (glycogen debranching enzyme (GDE) (**C**), glycogen branching enzyme (GBE) (**D**) and glycogen phosphorylase (GP) (**E**) in 1-, 10- and 30-min wash times to isolate diffusible proteins located within the muscle fibre. Data are expressed as means ± SD where the amount of protein diffused out into the wash solution is expressed as a percentage of the total (% of the wash and fibre (total)). *n* signifies the total number of fibre wash sets. Statistical comparison shown in table, one-way ANOVA within population, Bonferroni’s post hoc analyses to assess 1 vs 10 min and 10 vs 30 min timepoints (except GP, unpaired *t* test). Closed squares are T2D and open squares are CON

**Fig. 4:**
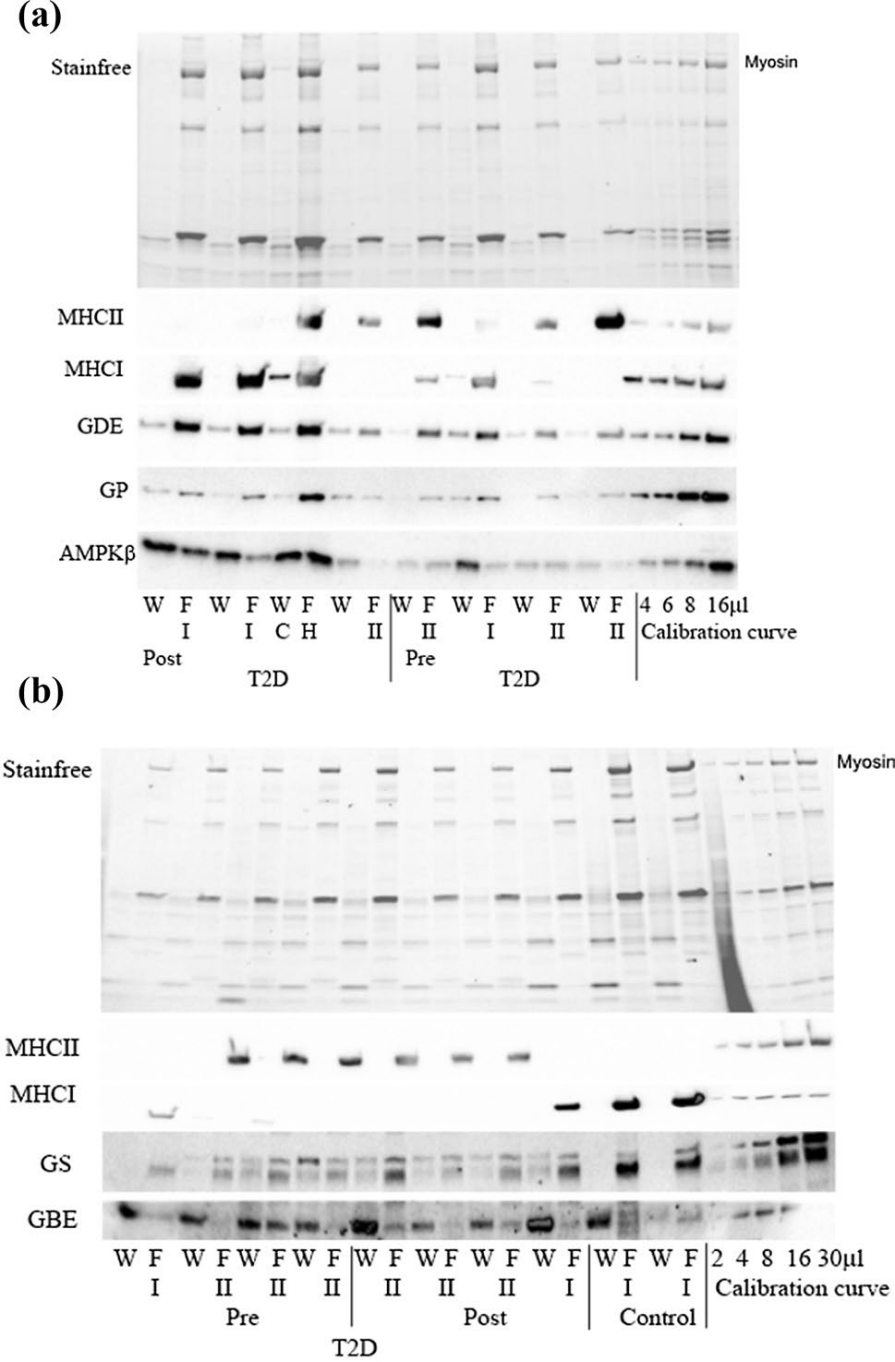
Representative Western blots of MHCI, MHCII, GDE, GP, AMPKβ, (**A**) and GS (lower band) and GBE (**B**) remaining within the fibre (F) or lost to the surrounding “wash solution” (W) after bathing individual mechanically skinned fibre segments in physiological intracellular solution for the indicated time. Each fibre segment was run with its corresponding wash solution in adjacent lane. StainFree pre-transfer image indicates the relative amount of tissue constituting the given muscle fibre segment, and the absence of MHC and actin signals in the W lanes shows there was little, if any, myofibril contamination in the wash solutions. Myosin heavy chain type indicated by I or II, C indicates contamination. A mixed sample calibration curve indicates sample detection limitations

Muscle fibres were dissected from freshly obtained muscle biopsies from CON and T2D and the proportion of a protein in the cytosol following 1-, 10- and 30-min washes measured by Western blotting, where wash and fibre pairs were analysed side by side. To examine the diffusion time course, Type I and Type II fibres were pooled (except for GDE). In CON, the appearance of a protein was not different between 1- and 10-min washes, with more appearing in the wash at 30-min, compared with 10-min, for AMPK (Fig. 3). In T2D, comparing 1- and 10-min washes, there was more AMPK and GP in the 10-min wash with the appearance of a protein not different between 10- and 30-min washes (Fig. 3). Comparing fibre types across CON and T2D, apart from GDE in Type I fibres, there was a greater abundance of glycogen-related proteins located in the cytosol in fibres from T2D compared to CON individuals, in both Type I and Type II fibres (Fig. 4, Table 1). The hyperinsulinaemic, euglycaemic clamp made no significant difference to the diffusible portion of the fibres for any of the glycogen-related proteins. The diffusibility of AMPK-β2 was not influenced by type 2 diabetes or the hyperinsulinaemic, euglycaemic clamp (Fig. 4 and Table 1).

**Table 1:** Proportion of diffusible protein in Type I and Type II muscle fibres from control and T2D individuals, and before (Pre) and after (Post) a 1 h hyperglycaemic, euglycaemic insulin clamp in T2D

	Control (mean ± S.D.)		Type 2 Diabetic (mean ± S.D.)			
	Type I	Type II	Type I		Type II	
			Pre	Post	Pre	Post
GDE (% of total)	18 ± 24 *n* = 26/6	15 ± 22 *n* = 15/6	18 ± 19 *n* = 24/8	24 ± 27 *n* = 22/7	30 ± 24*^,$^ *n* = 31/8	36 ± 27 *n* = 27/8
GBE (% of total)	47 ± 40 *n* = 21/6	45 ± 30 *n* = 13/6	71 ± 24* *n* = 21/8	73 ± 20 *n* = 22/8	69 ± 27* *n* = 38/8	72 ± 31 *n* = 25/8
GP (% of total)	26 ± 25 *n* = 19/6	26 ± 14 *n* = 10/6	44 ± 31* *n* = 31/8	44 ± 28 *n* = 18/8	45 ± 28* *n* = 29/8	45 ± 22 *n* = 25/8
GS (% of total)	4 ± 8 *n* = 17/6	1 ± 1 *n* = 8/6	18 ± 22* *n* = 20/7	17 ± 25 *n* = 19/8	17 ± 21* *n* = 20/ 8	21 ± 20 *n* = 19/7
AMPK-β2 (% of total)	68 ± 20 *n* = 31/6	73 ± 16 *n* = 16/6	72 ± 21 *n* = 28/8	72 ± 20 *n* = 27/7	64 ± 25 *n* = 34/8	56 ± 27 *n* = 30/8

Diffusion of glycogen-related proteins (glycogen debranching enzyme (GDE), glycogen branching enzyme (GBE), glycogen phosphorylase (GP), glycogen synthase (GS) and AMPK-β2 as measured using a 10-min wash time to isolate diffusible proteins located within the muscle fibre. Data are expressed as means ± S.D. where the amount of protein diffused out into the wash solution is expressed as a percentage of the total (% of the wash and fibre (total)). *n* signifies the total number of fibre wash sets/number of subjects. *P < 0.05 compared to matched fibre type from control subjects, $*P* < 0.05 compared to Type I fibres from T2D subjects, unpaired student t-test

### Glycogen amount and diffusibility in single fibres from control and T2D individuals.

**Fig. 5:**
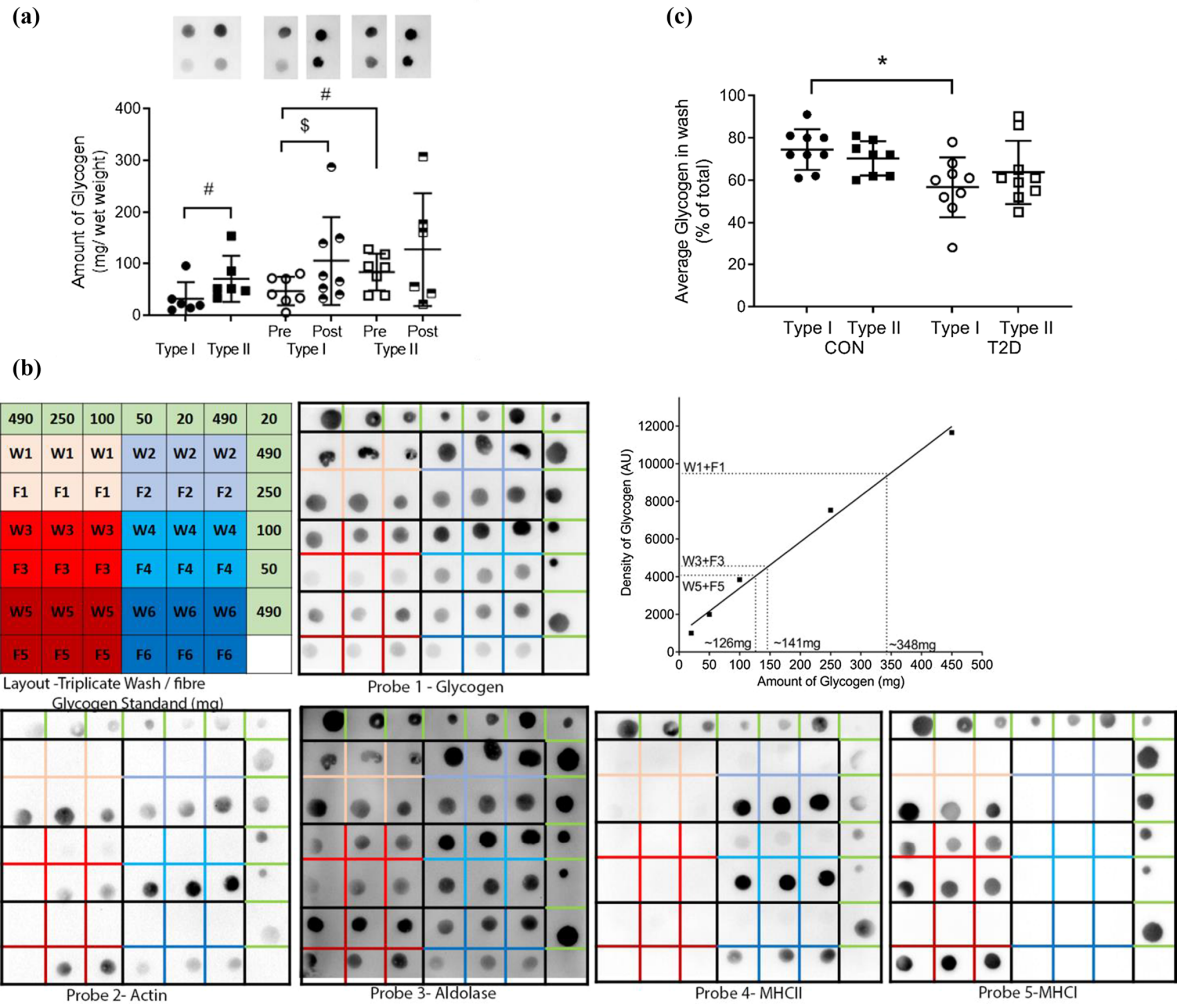
Fibre type dependence of glycogen content and diffusibility in control and T2D individuals. Amount of total and diffusible glycogen in Type I and Type II fibres isolated from control (CON) and T2D individuals**. A** Glycogen content in individual fibre types. Top, representative Dot Blot image indicating glycogen amount in the Type I and II fibres from the same CON or T2D individual (pre and post hyperinsulinaemic, euglycaemic clamp). Bottom, total glycogen for each group (mean + SD, *N* = 6–10). ^$^Indicates significant difference from Type I Pre, *P* < 0.05; # indicates *P* = 0.05 between Type I vs Type II CON. **B** A representative Dot Blot showing detection of proteins wash (W) and matched fibre (F) in triplicate and a standard curve of a known amount of Glycogen (green boxes), with each amount run in at least duplicate. In the wash, (aldolase as the control) and fibre (actin as the control and MHCI and II to identify fibre type) using specific antibodies. Glycogen can be seen in both the wash and fibres. Each fibre and wash set run in triplicate (denotes F and W seen in top left 2X3, each set shown in separate colour). No stripping of membranes between probes and residual actin seen in aldolase probe. Quantification of glycogen in MHCI and MHCII fibres using the calibration curve derived by plotting the band density for each glycogen sample (see Dot blot Probe 1 glycogen) against the amount of glycogen. Total glycogen of wash and fibre (W1 + F1 ~ 350 mg, W3 + F3 ~ 141 mg and W5 + F5 ~ 126 mg) derived using the calibration curve to convert average band intensity for the triplicate fibre sample into the amount of glycogen present. **C.** Percentage of diffusible glycogen in Type I and Type II fibres isolated from CON and T2D individuals. (mean + S.D.) where the amount of protein diffused out into the wash solution is expressed as a percentage of the total. *N* = 8–9 individuals per group * indicates *P* < 0.05 compared to matched fibre type from control subjects, Tukey multiple comparison

In the basal (non-insulin stimulated) state, there was more glycogen in Type II compared with Type I fibres in CON (*P* = 0.05) and T2D (*P* < 0.01, Fig. 5A). There was no difference in the abundance of glycogen between either Type I or Type II fibres in CON and T2D. Following the insulin clamp there was no difference in the abundance of glycogen between Type I and Type II fibres, although the high insulin insult during the clamp resulted in a significant increase in the amount of glycogen in Type I fibres compared with Type I fibres before the clamp (*P* < 0.001). The glycogen diffusibility method was validated with the observation of the cytosolic enzyme aldolase appropriately localised in the diffusible pool and both the myofibrillar proteins actin and the fibre specific MHC I and MHC II proteins in the fibre pool, as seen in Fig. 5B. To identify the proportion glycogen in each pool the amount of glycogen in the diffusible (wash) pool was expressed as a percentage of the total (i.e., glycogen in wash and fibre, Fig. 5C). The majority of glycogen was diffusible in all fibres. It was 70% and 65% for Type I and Type II fibres, respectively in CON, and 53% and 60% for Type I and Type II fibres, respectively, in T2D. A smaller subset of fibres were assessed for glycogen diffusibility with 1- and 30-min washes, which were not different from the 10-min washes within either T2D or CON (Fig. 3). This was different in Type I fibres from T2D compared with CON (*P* < 0.005). The amount of diffusible glycogen was not altered after insulin clamp (results not shown).

## Discussion

The major findings of this paper are that the majority of glycogen is cytosolic and that the distribution of glycogen-related proteins in the cytosol was greater in T2D compared with CON. Given that type 2 diabetes manifests because of poor glucose regulation and that fibre type distribution is altered in T2D, we addressed important fibre specific differences in skeletal muscle glycogen and related proteins. A lower proportion of Type I fibres in T2D than CON (Fig. 1; [9, 31]) may explain reported alterations in metabolic capacity in type 2 diabetes individuals [32]. The differential distributions of key glycogen-related proteins between Type I and Type II (constituting a mixture of fibres expressing MHC IIa and MHCIIa/IIx, but not pure MHCIIx) muscle fibres observed in CON was not reflected in T2D, identifying a potential point of dysregulation.

Glycogen was higher in Type II compared with Type I muscle fibres in both CON and T2D, in concordance with previous fibre-specific comparisons [9]. This is despite glucose being locked inside a muscle cell through the action of intracellular hexokinase, which is ~ 4–fivefold higher in Type I compared with Type II fibres from both healthy and type 2 diabetes individuals [9]. This suggests that hexokinase does not directly influence glycogen content, but rather presents a higher glucose handling ability in oxidative fibres during periods of high circulating glucose. There was no difference in the glycogen content between Type I or Type II fibres in CON and T2D, similar to a comparison of glycogen in whole muscle [33]. This does not support the suggestion that there is a reduced glycogen storage capacity in type 2 diabetes due to a stronger feedback inhibition of glycogen synthase [34]. Our findings show similar skeletal muscle glycogen content between healthy individuals and those with type 2 diabetes despite the difference in basal blood glucose levels. This likely also reflects the fibre type shift seen in muscle from type 2 diabetes, which we and others have shown [9]. After the hyperinsulinaemic, euglycaemic clamp in T2D, glycogen content increased in Type I but not Type II fibres, although there was no difference in the abundance of GS protein, like others who reported no change in the various phosphorylation states of GS [9]. Whilst increased glycogen content has been positively correlated with GS activity [35–37], these data suggest the presence of additional regulatory mechanisms. To address this, we investigated the abundance of the various glycogen-related proteins and their intracellular location before and after a hyperinsulinaemic, euglycaemic clamp.

The greater abundance of the glycogen catabolic enzyme, GP, in Type II compared with Type I fibres from our CON was similar to that reported for young, healthy humans [38] as well as GP activity [39]. GP was ~ threefold greater in both Type I and Type II in T2D compared with CON (Fig. 2B), indicative of an enhanced capacity for cleavage of alpha-1,4-linkages of glycogen. GDE, which is necessary for cleavage of alpha-1,6-linkages breakdown, may be rate limiting as there was no concomitant difference in its abundance. Some of this was seemingly compensated for following the hyperinsulinaemic, euglycaemic clamp, when the abundance of GDE increased ~ 1.4–1.8-fold in both fibre types in T2D. This may be due to an effect of the higher circulating insulin on the protein stability, i.e., altered protein degradation or increased half-life of GDE. This is plausible given that in isolated rat diaphragm, insulin stimulated protein synthesis and inhibited protein degradation, albeit with supraphysiological insulin levels [40]. Nevertheless, the increased GDE abundance suggests that glycogen was more accessible as a fuel source under this heightened insulinaemic and normal blood glucose environment.

After initiation, the synthesis of glycogen granules relies on the actions of GBE and GS. Overall, there was little difference in the abundance of these proteins when comparing either fibre type or CON vs T2D. There was, however, about half as much GBE in Type II compared to Type I muscle fibres in T2D which was not evident in CON (Fig. 4D). An implication of having reduced GBE in Type II fibres may be a reduced ability for the glycogen granules to be assembled which may contribute somehow to impaired glucose regulation. This is in part supported by the observations following the normalisation of blood glucose with the hyperinsulinaemic, euglycaemic clamp, where there was an increase in GBE in Type II fibres, suggesting either an increase in synthesis or decrease in degradation of that protein. Albers and colleagues also reported no fibre type difference in these proteins in muscle from type 2 diabetes individuals, however, they reported GS higher in Type I compared with Type II fibres from control individuals [9]. It is unclear why this discrepancy presented, however, it should be noted that changes in the protein alone are insufficient to identify functionally relevant findings. This is particularly relevant for GS, whose activity is highly regulated via one of its nine phosphorylation sites or independently of phosphorylation via allosteric regulation activated by glucose-6-phosphate.

The major AMPK β-isoform in human skeletal muscle, AMPK-β2, showed no fibre type differences (Fig. 2J). Supporting our findings, in young healthy individuals others have found no fibre type difference in AMPK-β2, although there was a greater abundance of AMPK-β1 in Type I compared with Type II fibres [41]. Previous human studies have investigated AMPK-β1/β2 amounts in muscle homogenates collected from type 2 diabetes and control individuals and found no difference in either AMPK-β1 [42] or AMPK-β2 [20, 43]. Nevertheless, in our study Type II fibres from T2D had less AMPK-β2 than those from CON suggesting that the dysregulation in glucose uptake that occurs in type 2 diabetes may be in part due to AMPK abundance and potential activity in a fibre specific manner. Given the shift to an increased proportion of Type II fibres in T2D compared with CON (Fig. 1) and that AMPK-β2 is more abundant in those Type II fibres from CON individuals, it is perhaps not surprising that muscle homogenates are unable to identify differences in AMPK-β2 between control and type 2 diabetes individuals. AMPK-β has been shown to be associated with a number of key mechanisms of glucose regulation, including the activation of GS activity [44], GLUT4 translocation [45, 46] and the regulation of lipid oxidation through targeting acetyl-CoA carboxylase activity (ACC) [47]. This means that any reduction in the expression of AMPK-β2 in Type II fibres in type 2 diabetes would likely reflect a decreased glucose handling capability in skeletal muscle.

### Diffusibility

The various glycogen-related proteins had a strikingly higher cytosolic or loosely bound abundance in both Type I and II fibres in T2D compared with CON. The ramification of this could be dysregulation of the glycogen granule due to altered protein–protein interactions changing the rate of glycogen granule formation and breakdown. This would alter the availability of readily accessible energy with implications for glycogen use in muscle of type 2 diabetes individuals. Interestingly, there was no such differential between CON and T2D fibres in the predominant cytoplasmic localisation of AMPK-β2. We have previously demonstrated that in rat skeletal muscle AMPK-β2 was not associated with the glycogen granule, despite the carbohydrate binding motif [21]. This is likely also the situation in human skeletal muscle suggesting that the 25% AMPK-β2 that is bound in fibres is localised to regions other than glycogen and is likely unrelated to glycogen regulation.

### Glycogen

A major finding of this study is the first evidence we provide for a considerable pool of diffusible glycogen, indicating a novel cytosolic or loosely bound pool in human skeletal muscle. This large pool amounted to 47–52% and 50–70% of the total glycogen in muscle after 1- and 10-min washes, respectively. This is perhaps surprising based on electron microscopy images that show glycogen granules tightly embedded within/between various cellular structures [48]. Our findings are validated by the presence and absence of cytosolic (aldolase) and cytoskeletal (actin) control proteins in the expected locations. Further, glycogen granules are ~ 25 nm in diameter on average (range 10–44 nm) [49] being much larger than proteins, where a 50 kDa protein is predicted to be ~ 2.4 nm. Appearance of glycogen in the 1-min wash confirms a cytosolic location, similar to that shown for proteins such as heat shock proteins [25] and calpains [26]. Importantly, the robustness of this technique is demonstrated where the cytosolic localisation of AMPKβ2 was altered following in situ muscle stimulation of rat muscle [21]. The proportion of diffusible glycogen in Type I fibres was less in T2D compared with CON (Fig. 5C). Using a similar approach to that used here, > 75% of total glycogen was reported to be diffusible in toad muscle [50]. Whilst distinct pools of glycogen have been identified using isolated human single skeletal muscle fibre segments, with most glycogen within the intermyofibrillar followed by intramyofibrillar and subsarcolemmal spaces [48], our findings add a new pool that amounts to over half the total glycogen, and highlight an important limitation when interpreting electron microscopy studies. The reason for this is likely because sample preparation for electron microscopy requires washing and fixation steps that would either wash away this newly identified diffusible pool or render it an artificial location in the muscle fibre following fixation steps. Others have reported two distinct fractions of glycogen according to their solubility by either acid hydrolysis or enzymatic hydrolysis [51, 52], however, it is unlikely one of these represents the cytosolic pool of glycogen isolated here using physiologically relevant conditions. Finally, we considered if differing glycogen granule sizes, which can be 10–56 nm [53, 54], could be a determinant of cytosolic location, although considered this unlikely because glycogen granule size has been shown to be normally distributed between 10 and 44 nm with no distinctive pools detected [53]. This poses a limitation of our study in that we were not able to investigate the size of the glycogen granules within the diffusible subcellular pool, as to do so requires electron microscopy steps, which means that even if the granules could be captured in the muscle fibres, the diffusible glycogen could not be distinguished from other glycogen granules.

## Conclusions

**Fig. 6:**
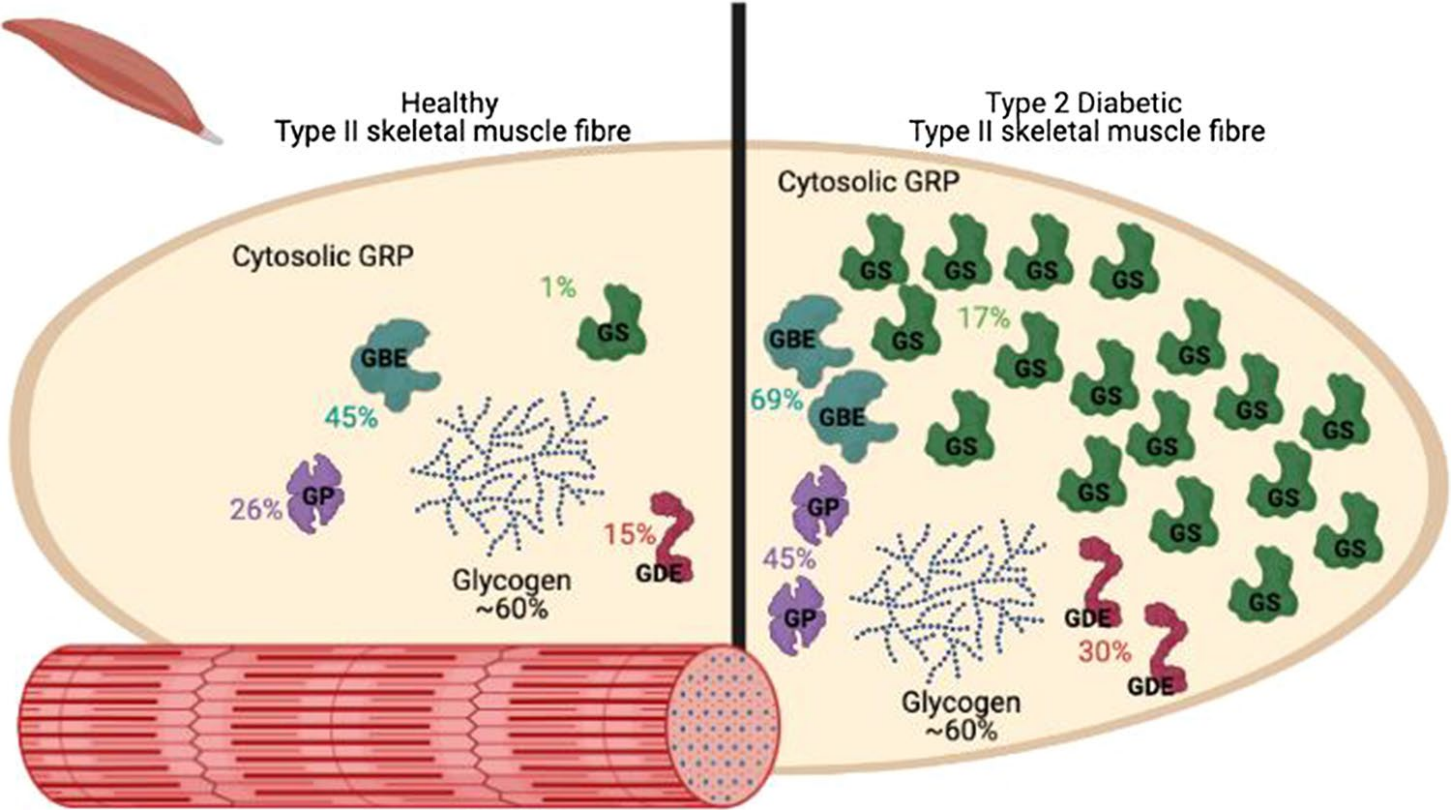
Graphical depiction of fold differences of glycogen-related proteins in the cytosol of Type II skeletal muscle cells afflicted with T2D*.* Although the ~ 60% cytosolic glycogen does not change there is a dramatic increase in the amount of diffusible GRP compared to amounts in healthy fibres

We show the potential to anabolise glycogen granules depends on whether Type I or Type II fibres are examined, and this proportion is different in the muscle from people with type 2 diabetes. Interestingly, AMPK-β2 showed little differences in the muscles and fibre types examined, and it is warranted to examine fibre type-specific AMPK activity in muscle from healthy control and type 2 diabetes individuals to ascertain whether AMPK can play a major role in glycogen regulation. Protein function is dependent on location within a cell, and we have shown that proteins vital for glycogen regulation, either through synthesis or degradation, are differentially localised to the cytosol in muscle from control and type 2 diabetes individuals. We have derived a model from our findings, shown in Fig. 6, depicting the difference in relative abundance of cytosolic or loosely bound proteins in muscle from control individuals and type 2 diabetes patients. Further exploration is required to ascertain if they occur in the pre-diabetic stage, or after type 2 diabetes is present, and thereby reveal potential mechanisms for targeting in the treatment of type 2 diabetes.

## Data Availability

The data that support the findings of this study are available from the corresponding author upon reasonable request.
